# Oral contraceptives and antibiotics. A cross-sectional study about patients’ knowledge in general practice

**DOI:** 10.1186/s12978-015-0037-4

**Published:** 2015-05-14

**Authors:** Kathryn Hoffmann, Aaron George, Lukas Heschl, Anna Katharina Leifheit, Manfred Maier

**Affiliations:** Department of General Practice and Family Medicine, Centre for Public Health, Medical, University of Vienna, Kinderspitalgasse 15/I 1st floor, 1090 Vienna, Austria; Department of Community and Family Medicine, Duke Medical Center, 2301 Erwin Rd, Box 3886, Durham, 27705 USA North Carolina

**Keywords:** Antibiotic interaction, Oral contraceptive pill, Knowledge, Health literacy, Primary care, Family planning, Austria

## Abstract

**Background:**

The evidence regarding oral contraceptives and its effectiveness with concomitant ingestion of antibiotics is conflicting. Until evidence becomes clearer, patients should be aware of this possible interaction. The aim of this study was to assess the knowledge and the source of information about this interaction in GP patients in Austria.

**Methods:**

Within the framework of the APRES study, 20 Austrian GPs were purposefully selected from among a GP research network and were asked to recruit 200 patients each. The patient cohort was asked to complete a questionnaire. Subsequent analysis included descriptive statistics, statistical tests and logistic regression models.

**Findings:**

Overall, 3280 questionnaires could be used for analysis. Of these, 29.7 % (n = 974) of patients acknowledged an awareness of the interaction of antibiotics with OCPs. Women under the age of 46 years acknowledged this interaction in 52.3 % of cases. Positive associations for the belief in an existing interaction in women were identified with age (OR 2.2) and having read the package inserts (OR 1.6). Further, belief was recognized in males based on age (OR 2.5) and tertiary education (OR 2.0). The main source of information regarding antibiotics was the GP (55.9 %).

**Conclusions:**

Less than one-third of all participants and half of the women in the reproductive age acknowledged an interaction between antibiotics and OCPs. Since the GP is the main source of information, this finding depicts a large potential for knowledge transfer within the primary health care setting. A multifaceted strategy is needed at both the population and the GP level to improve awareness and to address these educational gaps.

## Introduction and background

Oral contraceptive pills (OCP) and antibiotics are both among the most frequently prescribed drugs in the United States and Europe, including Austria [1, 2]. However, current evidence regarding the intake of antibiotics and any potential impact on the effectiveness of the OCP is conflicting. While a large epidemiological U.S. study concluded that no association between concomitant antibiotic use and the risk of breakthrough pregnancy among OPC users could be found [3], a case-crossover study from the Netherlands reported that a relationship could be likely [4]. Further, results of pharmacokinetic and clinical studies appear to be contradictive: in some studies a possible effect of impairment of the enterohepatic circulation by contraceptives and antibiotics was witnessed [5, 6], while in others oral antibiotics did not affect the pharmacokinetics of OCPs (with the exception of rifampin) [7]. Dickinson *et al.* reported that individual patients on certain antibiotics did show substantial decreases in the plasma concentrations of ethinyl estradiol, although no systematic interaction between antibiotics and OPCs could be found. As it is difficult to identify these women in advance of complications, the authors recommended a cautious approach [8]. Until the evidence is clear, this approach seems reasonable and the possible interactions should continue to be included among the package inserts of both antibiotics and OCPs.

Additionally, recent health policy publications have recognized a prominent role for “health literacy” as the “*people’s knowledge, motivation and competences to access, understand, appraise, and apply health information […]* [9]”. Considering this, citizens should be aware of conflicting fields in order to be able to appraise potential risk for their own situation and to actively participate in decision-making. Particularly, this is of high relevance in the field of family planning because of the social and psychological consequences of unintended pregnancies for the mother, father, and the child [10, 11].

The knowledge about drug interactions has been observed as low among the general population, although, adverse drug events are the most common type of injury occurring in patients [12–15] and the public has expressed interest in greater awareness of these medications [14].

The present study - intended to function as an add-on to the European APRES project [16]- aimed for a two goal approach:

- First, we aimed to assess the knowledge about possible drug interactions in both men and women. Inclusion of both genders was an important element, as both might fill the role of parent, partner or friend, and may influence women that take OCPs. Further, it is recognized that social and cultural context has a high impact on individuals and their willingness to learn about or change their situation if offered the intrinsic or extrinsic opportunity to do so [9, 17]. Cultural and social beliefs and norms are often driven by societal expectations, and can be particularly influenced by the literacy of a husband, more than the knowledge of a woman, in patriarchal cultures [18, 19].
- The second aim was to analyse possible social and cultural factors associated with patient knowledge. Further, the main source of a given patient’s information on knowledge about antibiotic interactions as well as other possible influencing factors from the GPs’ side were assessed.

## Methods

### Design

We performed an add-on study to the European APRES project, a cross-sectional study that lasted from November 2010 until July 2011 [16]. More detailed information concerning the design and the overall aim of the APRES project are described in the publication by van Bijnen *et al.* [16].

In Austria, a stratified sample of 20 purposefully selected GPs was recruited via electronic invitations through the Austrian Society of General Practice, the research network of the Department of General Practice at the Medical University of Vienna and personal contacts. Each GP was asked to complete a short questionnaire (“GP questionnaire”) concerning demographic variables and the location of their practice. The GPs were asked to recruit 200 consecutive patients from within their practices during consultation hours. After signing an informed consent form, patients were asked to complete two questionnaires (“patient questionnaires”). Following the APRES study protocol, patients younger than four years, patients with infections or symptoms of an infective disease, patients treated with antibiotics within the last three months, patients with terminal diseases, patients with immunosuppressive diseases or treatments, patients who had been hospitalized within the last three months and patients living in a nursing home were excluded. In addition, for this analysis we included only patients aged 12 years and older because the drug interaction question was less relevant for those younger patients.

## Questionnaires

### Patient questionnaire

For this add-on study, an additional 12 item questionnaire was developed which was handed out alongside the APRES patient questionnaire. In this questionnaire, six questions covered knowledge about antibiotics, one of which specifically addressed the antibiotic and OCP interaction. The detailed add-on questions can be viewed in a separate publication [20].

The question related to the antibiotic to OCP interaction is as follows: “Taking antibiotics can lead to ineffectiveness of contraception by the Pill.” There were three response options: “yes”, “no” and “I don’t know”. Later, for the binary logistic regression model this variable was dichotomized into the answer option “yes”, versus the other two options. The interaction variable was defined as a dependent variable.

The demographic data requested and relevant for this analysis included the educational level and country of origin of the patients. Highest educational level accomplished was assessed in three categories: primary education, secondary education (apprenticeship or secondary school) and tertiary education (university or any further education). Migration status was assessed with the question “What is your country of origin?” This variable was stratified within three categories: Austria, other European Union (EU) 28 countries as well as Norway and Switzerland (EU28+), and “all other countries”. Finally, the main source of information related to antibiotics was asked: GP, specialist, pharmacy, media, internet, package insert, own estimation or relatives and friends. Respondents were instructed to mark only one out of the eight answer categories listed. The answer categories were then clustered into GP, specialist/pharmacy, media/internet/relatives/friends/own estimation, and package insert.

In addition, other socio-demographic data from the APRES patient questionnaire, such as gender and age of the patients, was taken into account. The age of patients was clustered into seven groups, namely those up to the age of 25, those aged 26–35 years, 36–45 years, 46–55 years, 56–67 years, 66–75 years and participants aged 76 years and older. Moreover, the age of the participants was dichotomized into an age group of persons younger than 46 years of age and those older than 46 years of age. This dichotomy was assessed to identify women in the reproductive age and to have a direct comparison with the male group of the same age.

### GP questionnaire

From the GP questionnaire the gender and location (urban or rural area) of the GPs was used for analysis. Additionally, the identification code of the GP offices was used for the regression model to adjust for the clustering of patients in GP practices.

The demographic variables from both patient and GP questionnaire were defined as explanatory variables.

### Data analyses

First, the sample of patients and GPs were characterized using descriptive methods. Tests for subgroup analyses were performed by means of cross-tabs. The statistical test applied was the Chi-Square Independency test. If an independency could not be proven, the z-test, including the Bonferroni method for multiple testing, was applied to identify those sub-groups which were dependent. The significance level for all calculations was p < 0.05 and the confidence interval 95 %.

A multivariable logistic regression model was employed for the women’s and men’s group separately in which the interaction knowledge variable “yes” was defined as the dependent variable. All demographic variables and the GP identification code variable were taken into the model simultaneously. The results of all regression models are presented as odds ratios with 95 % confidence intervals. Nagelkerkes’ R^2^ (logistic regression models) is presented as a measure of model-fit.

SPSS Statistics 20.0 was used for all statistical analyses.

### Ethical considerations

For those participants younger than 18 years of age, the parent and adolescent each completed written informed consent forms separately. The study and analysis was approved by the Ethics Committee of the Medical University Vienna (EC # 568/2010).

## Results

### Sample and source of information

Overall, 3280 questionnaires could be used for analysis. A detailed description of GPs and patients included in this study has been published [20]. The patients included were 56.4 % women; the primary level of education was 49.0 %, while the secondary and tertiary levels were 37.3 13.7 % respectively. The country of origin was found to b86% Austria and 6.0 % EU28+ country. Age groups were distributed as follows: age group 12–25 years 11.7 % females (f) and 13.0 % males (m), age group 26–35 years 15.1 % f and 13.7 % m, age group 36–45 years 18.3 % f and 15.6 % m, age group 46–55 years 19.4 % f and 21.4 % m, age group 56–65 years 14.0 % f and 16.9 % m, age group 66–75 years 15.2 % f and 14.6 % m, and age group 76 years and older 6.3 % f and 4.8 % m. The participating GPs were 30.0 % women and the location of the office was 55.0 % in rural areas.

Regarding the source of information about antibiotics, 55.9 % indicated that this came from the GP, 3.6 % from specialists/pharmacy, and 28.3 % from media/internet/friends/relatives/ neighbours/own estimation. The final 12.0 % related that the package insert was the primary source of information about antibiotics.

### Antibiotics- OCP interaction

When questioned about awareness of interaction between antibiotics and OCPs, 29.7 % (n = 974; f 31.1 % vs. m 19.9 %; p < 0.05) of participants marked “yes”, while 9.0 % (n = 296; f 10.2 % vs. m 7.6 %; p < 0.05) indicated “no”, 59.3 % (n = 1946; f 51.4 % vs. m 69.7 %;p < 0.05) “not known”, and 2.0 % (n = 64; f 1.3 % vs. m 2.7 %; p < 0.05) of the participants did not respond to the question.

Within the group of the women in the reproductive age (those below 46 years of age) (n = 801), 52.3 % marked “yes”, 11.8 % “no” and 35.9 % marked “don’t know/no answer”. In contrast, within the group of women 46 years and older (n = 977) 24.4 % marked “yes”, 9.0 % indicated “no and 66.6 % marked “don’t know/no answer”. Males younger than 46 years of age (n = 586) marked “yes” 30.4 % of the time, while 10.1 % of this same group marked “no” and the final 59.6 % indicated “don’t know/no answer”. Finally, among men 46 years and older (n = 794) 12.1 % indicated “yes”, 5.9 % “no” and 82.0 % marked “don’t know/no answer”.

Table 1 shows the distribution of responses to the question on drug interaction in relation to the patients’ demographic variables, the sex of the GP, the location of the GP office, and the source of information related to antibiotics for women. Table 2 shows the same results for male patients. In addition, statistically significant differences for subcategories of the variables are shown.

**Table 1:** Female subgroup analysis related to the antibiotics-OCP interaction knowledge

				Yes (%)	No (%)	Dont know/no answer (%)	*p*-value
Sex	Age group	Categories	Subcategories (n)				
F	<46 years	Age groups (years)					<0.001
			−25 (n = 210)	58.1	13.8	28.1	
			26-35 (n = 270)	58.9	10.4	30.7	
			36-45 (n = 325)	43.1	11.7	45.2	
	46+ years						<0.001
			46-55 (n = 345)	36.8	12.2	51.0	
			56-65 (n = 252)	23.8	9.9	66.3	
			66-75 (n = 269)	15.6	5.2	79.2	
			76+ (n = 111)	8.1	6.3	85.6	
	<46 years	Educational level					0.029
			Primary (n = 299)	50.8	10.0	39.1	
			Secondary (n = 354)	55.6	10.5	33.9	
			Tertiary (n = 148)	46.6	18.9	34.5	
	46+ years						0.013
			Primary (n = 532)	24.8	7.3	67.9	
			Secondary (n = 318)	23.9	8.8	67.3	
			Tertiary (n = 112)	23.2	17.9	58.9	
	<46 years	Country of origin					<0.001
			Austria (n = 678)	55.9	10.8	33.3	
			EU 27 + (n = 51)	41.2	17.6	41.2	
			Others (n = 74)	27.0	17.6	55.4	
	46+ years						0.274
			Austria (n = 857)	24.5	8.5	67.0	
			EU 28+ (n = 55)	16.4	14.5	69.1	
			Others (n = 64)	29.7	10.9	59.4	
	<46 years	GP gender					0.015
			Female GP (n = 207)	44.4	11.6	44.0	
			Male GP (n = 598)	55.0	11.9	33.1	
	46+ years						0.080
			Female GP (n = 338)	20.1	9.5	70.4	
			Male GP (n = 639)	26.6	8.8	64.6	
	<46 years	Location GP office					<0.001
			Urban (n = 340)	44.7	15.3	40.0	
			Rural (n = 465)	57.8	9.2	32.9	
	46+ years						0.002
			Urban (n = 481)	20.2	11.2	68.6	
			Rural (n = 496)	28.4	6.9	64.7	
	<46 years	Source of information					0.580
			GP (n = 338)	50.9	10.1	39.1	
			Specialist/pharmacy (n = 26)	57.7	11.5	30.8	
			Media/Internet/Friends/Own (n = 157)	52.9	11.5	35.7	
			Package insert (n = 123)	61.0	9.8	29.3	
	46+ years						0.035
			GP (n = 496)	19.2	8.9	72.0	
			Specialist/pharmacy (n = 25)	16.0	8.0	76.0	
			Media/Internet/Friends/Own (n = 212)	26.4	8.5	65.1	
			Package insert (n = 97)	29.9	14.4	55.7	

Significance at a significant level of p < 0.05

**Table 2:** Male subgroup analysis related to the antibiotics-OCP interaction knowledge

				Yes (%)	No (%)	Dont know/no answer (%)	*p*-value
Sex	Age group	Categories	Subcategories (n)				
M	<46 years	Age groups (years)					0.001
			−25 (n = 180)	37.2	11.7	51.1	
			26-35 (n = 190)	33.7	11.6	54.7	
			36-45 (n = 216)	21.8	7.4	70.8	
	46+ years						0.001
			46-55 (n = 293)	18.4	7.2	74.4	
			56-65 (n = 235)	9.8	6.0	84.3	
			66-75 (n = 200)	8.0	5.5	86.5	
			76+ (n = 60)	4.5	1.5	93.9	
	<46 years	Educational level					0.035
			Primary (n = 248)	24.2	12.1	63.7	
			Secondary (n = 257)	33.9	9.7	56.4	
			Tertiary (n = 80)	38.8	5.0	56.3	
	46+ years						0.071
			Primary (n = 454)	11.2	4.4	84.4	
			Secondary (n = 239)	11.3	7.5	81.2	
			Tertiary (n = 93)	17.2	9.7	73.1	
	<46 years	Country of origin					
			Austria (n = 486)	31.3	9.3	59.5	
			EU 27 + (n = 36)	36.1	11.1	52.8	
			Others (n = 61)	21.3	16.4	62.3	
	46+ years						0.314
			Austria (n = 686)	12.2	5.8	81.9	
			EU 27 + (n = 51)	17.6	5.9	76.5	
			Others (n = 51)	3.9	5.9	90.2	
	<46 years	GP gender					0.914
			Female GP (n = 116)	31.9	9.5	58.6	
			Male GP (n = 470)	30.0	10.2	59.8	
	46+ years						0.154
			Female GP (n = 214)	8.4	6.1	85.5	
			Male GP (n = 580)	13.4	5.9	80.7	
	<46 years	Location GP office					0.746
			Urban (n = 271)	31.4	10.7	57.9	
			Rural (n = 315)	29.4	9.5	61.0	
	46+ years						0.007
			Urban (n = 322)	8.1	7.5	84.5	
			Rural (n = 472)	14.8	4.9	80.3	
	<46 years	Source of information					0.242
			GP (n = 233)	29.6	8.2	62.2	
			Specialist/pharmacy (n = 21)	38.1	23.8	38.1	
			Media/Internet/Friends/Own (n = 167)	30.5	9.6	59.9	
			Package insert (n = 55)	25.5	9.1	65.5	
	46+ years						0.002
			GP (n = 394)	14.5	2.3	83.2	
			Specialist/pharmacy (n = 23)	4.3	4.3	91.3	
			Media/Internet/Friends/Own (n = 209)	12.0	9.6	78.5	
			Package insert (n = 45)	4.4	4.4	91.1	

Significance at a significant level of p < 0.05

### Associations between the antibiotics- OCP interaction knowledge and demographic factors

Table 3 illustrates the results of the adjusted regression models for the group of women below as well as the group over 46 years of age, and the associations between their knowledge about the drug interaction and patient-factors, GP-factors, and source of information. Similarly, Table 4 shows these findings for males.

**Table 3:** Results of the adjusted regression model for women

		Women <46 years			Women 46 years and older		
Category	Subcategory	OR	CI 95 %	*p*-value	OR	CI 95 %	*p*-value
Educational level	Primary	1.0			1.0		
	Secondary	1.2	0.8-1.7	0.331	1.1	0.8-1.6	0.580
	Tertiary	1.1	0.6-1.7	0.961	0.8	0.4-1.4	0.391
Country of origin	Austria	1.0			1.0		
	EU 27 + EFTA	0.6	0.3-1.2	0.122	0.6	0.3-1.4	0.251
	Others	0.2	0.1-0.5	<0.001	1.4	0.7-2.7	0.353
Age groups(years)	−25/46-55	1.8	1.2-2.6	0.004	6.0	2.7-13.7	<0.001
	26-35/56-65	2.2	1.5-3.2	0.001	3.5	1.5-8.2	0.004
	36-45/66-75	1.0			2.4	1.1-5.7	0.042
	/76+				1.0		
GP gender	Female	0.8	0.5-1.3	0.339	0.8	0.5-1.2	0.291
	Male	1.0			1.0		
Location of GPoffice	Urban	0.7	0.5-1.1	0.105	0.8	0.5-1.2	0.291
	Rural	1.0			1.0		
Source ofinformation	GP	1.0			1.0		
	Specialist/pharmacy	1.5	0.6-3.5	0.380	0.7	0.2-2.1	0.516
	Media/Internet/Friends/Own estimation	1.2	0.8-1.9	0.308	1.6	1.1-2.3	0.030
	Package insert	1.6	1.1-2.6	0.031	1.5	0.9-2.6	0.103
Nagelkerkes R^2^				0.117			0.097

Adjusted for GP cluster (GP identification code)

Significant at a level of p <0.05

**Table 4:** Results of the adjusted regression model for men

		Men 45 years and younger			Men 46 years and older		
Category	Subcategory	OR	CI 95 %	*p*-value	OR	CI 95 %	*p*-value
Educational level	Primary	1.0			1.0		
	Secondary	1.5	0.9-2.3	0.088	1.1	0.6-1.8	0.955
	Tertiary	2.0	1.1-4.0	0.038	2.4	1.1-5.1	0.023
Country of origin	Austria	1.0			1.0		
	EU 27 + EFTA	1.6	0.7-3.5	0.283	0.9	0.3-2.2	0.820
	Others	0.5	0.2-1.0	0.053	0.3	0.1-1.4	0.140
Age groups (years)	−25/46-55	2.5	1.5-4.2	0.001	3.7	1.1-12.5	0.037
	26-35/56-65	1.9	1.2-3.2	0.012	2.5	0.7-8.7	0.164
	36-45/66-75	1.0			1.9	0.5-6.8	0.340
	/76+				1.0		
GP gender	Female	1.2	0.7-2.0	0.624	1.1	0.5-1.9	0.963
	Male	1.0			1.0		
Location of GP office	Urban	0.9	0.6-1.6	0.995	0.4	0.2-0.8	0.009
	Rural	1.0			1.0		
Source of information	GP	1.0			1.0		
	Specialist/pharmacy	1.3	0.5-3.4	0.623	0.3	0.1-2.5	0.277
	Media/Internet/Friends/Own estimation	1.1	0.7-1.6	0.913	0.6	0.4-1.3	0.295
	Package insert	0.9	0.5-1.8	0.753	0.3	0.1-1.1	0.069
Nagelkerkes R^2^				0.087			

Adjusted for GP cluster (GP identification code)

Significant at a level of p <0.05

## Discussion

This study shows that less than one third of all participants (29.7 %) are aware of a possible interaction between antibiotics and OCPs.

This finding is worrying, particularly against the background of recent literature recommending to inform the population of possible interactions between antibiotics and OCPs. Further, the intake of both antibiotics and OPCs is high in Austria: in the year 2010, the antibiotic consumption in Austria was approximately 15 DDD per 1000 inhabitants per day and 60.7 % of women between 15 and 30 years, and 28.6 % of women between 30 and 45 years took OCPs [2].

On the other hand, however, it is remarkable that this knowledge exceeds that for the well-established therapeutic spectrum of antibiotics described in another publication, where only 28.1 % of individuals were aware that antibiotics do not kill viruses [20].

### Interaction knowledge

Considering the different patient groups, it is not surprising that about half of the group of women below 46 years of age indicated an awareness of possible interactions between antibiotics and OCPs (52.3 %). This is the group with the most contact to OCPs and, therefore, has the highest likelihood of previous exposure to information about this interaction. This finding is similar to the results from a 2002 U.K. study in which 64 % of OCP taking women aged 18 to 24 years answered that they knew about an interaction between antibiotics and OCPs [21].

Evaluating the age groups further, it became clear that those women aged 26 to 35 most often recognized this interaction (58.9 %). However, with every ten year increase in age, nearly 10 % fewer women expressed awareness of a possible interaction (Table 1).

Knowledge of this interaction was recognized in less than one third of males (30.4 % for males aged less than 46 years) and far less (12.1 %) in the group of men above the age of 46 (Table 2). Similar to responses in the female groups, the age of males does show impact on the likelihood of knowledge about interaction, with the youngest men most often aware of this possible interaction (37.2 %). This finding was consistent in the multivariable regression models in which younger age was positively associated with the knowledge in all patient groups (Tables 3 and 4).

In comparison to all other age groups, only younger males with tertiary education demonstrated more knowledge in both the univariate (38.8 %, Table 2) and the multivariable models (OR 2.0, Table 4). This might reflect that this group is, potentially, more involved in the process of family planning at this stage of their lives and, eventually, take a more participative and responsible role in family planning [22]. However, this would warrant further study.

On the other hand, the youngest group of women had the most “no” responses to the question of awareness of interaction, even when compared to the respective male subgroup (13.8 %). Only women with tertiary education marked “no” more often (17.9 %, Table 1). The findings of a German pilot-study point in a similar direction: while most women were confident of being knowledgeable about OCPs, many were unaware of their knowledge gaps [23]. Explanations for this unexpected result could be that the main OCP users were still too young to have completed their third level of education. Further, it was considered that women with tertiary education could possibly take OCPs less often, as they are commonly over the age of 35, which is a risk factor for taking less effective contraceptive methods [18, 24, 25]. In contrast, this finding could be due to a bias, as explained in the introduction section, the scientific evidence about the interaction effect is conflicting. Participants who know about this could have responded “no” because they have a more detailed knowledge of the circumstances of this issue. Regardless, more in-depth analysis with qualitative or quantitative methods, including a further question related to the contraceptive method used could be of benefit to better explain this result.

In addition, women below 46 years of age from other countries marked “yes” significantly less often than women from Austria or EU28+ countries with only 27.0 % doing so (Table 1). In the multivariable model, those patients originating from another country showed a negative association with an OR of 0.2 compared to Austrians (Table 3). This is a common finding in epidemiological studies related to health knowledge and in health literary [26, 27]. One reason could be the obstacle inherent in speaking and understanding the language in the respective country. To improve the situation, it would be necessary to provide target-group specific information, for example by inventing and implementing picture-based and computer-tailored educational programs [28].

When asked for the source of information on antibiotic – OCP interaction, 55.9 % of all patients marked the GP. Younger men and both younger and older women who marked the GP as their main source of information had the lowest number of “yes” answers compared to the other information groups (Table 1 and 2). After adjustment in the multivariable regression model, a positive association could only be found for reading the package insert for younger women (OR 1.6) and information via media/internet/friends for older women (OR 1.6) compared to the information via a GP (Table 3). This finding depicts a large potential for knowledge transfer within the primary health care setting. The result might be due to the lack of clear scientific evidence as well as the resulting uncertainty of GPs on how to advise their patients. However, it is known that the overall knowledge about antibiotics in Austria compared to other EU countries is low [20, 29], although there was some improvement observed between 2010 and 2013. It has been suggested that, until evidence is further clarified, women should be informed about this possible interaction with OCPs. Therefore, GPs should consider the possibility of interaction each time they choose to prescribe an antibiotic [21, 30, 31]. In a retrospective analysis, Mastrantonio et al., for example, showed that out of 100 female patients aged 15–39 to whom antibiotics were prescribed, the concomitant use of contraception was documented in only 3 % [32].

Our findings could suggest a large potential for improvement in primary care for knowledge transfer regarding drug interactions, as patient knowledge is low and the consequences are dangerous [12–15]. A Norwegian study, for example, described that older patients desire counselling on drug interactions [14]. Despite this, counselling is recognized as a time consuming task and should be acknowledged in the financing system of GPs.

### Strengths and limitations

One strength of this analysis is that patient-related factors were taken into consideration, as well as the addition of possible physician-related factors such as gender, location of office, and the main source of information for patients. The large sample size and the provision for different migration and age groups and educational levels similar to the Austrian population are further strengths of this study. Compared to the demographic distribution of all GPs with offices in Austria in the year 2011 (n = 6527; males 60.7 %, females 39.3 %, mean age 52.5 years) the numbers of the GPs in the sample size are similar. Moreover, compared with the Austrian population in the year 2011 (51.2 % females; mean age 43.9 years), the gender distribution of the study sample is similar; however, the mean age of the sample is higher.

The recruitment strategy of both GPs and patients is a limitation, although, it is a usual approach in General Practice research [33]. Due to the fact that the participation of GPs and patients was on a voluntary basis and not based on a randomized sample, it may be speculated that only those GPs and patients interested in the topic of antibiotic resistance participated in the study. This would lead to an over-estimation of the real knowledge.

Moreover, GPs did not identify how many patients refused to take part in this study.

Another important limitation is that we did not survey the prevalence of OCP intake among the participants and, therefore, no conclusions about the specific knowledge of the OCP user group can be drawn. However, this was not an aim of this study. Another methodological drawback is the fact that this study is cross-sectional and, therefore, of limited explanatory power. Furthermore, results are based on descriptive and self-reported survey data. A further limitation is that the questionnaires were available in German only. Lastly, the fact that the source of information was surveyed at the GP offices could have led to a selection bias, due to participants that marked the GP as their main source of information.

## Conclusion

Experts recommend informing OCP users of the potential for an interaction between antibiotics and OCPs and the risk on the effectiveness of OCPs. Clinicians are encouraged to advise female patients on the use of additional measures of birth control during and up to one week after antibiotic therapy [34]. Despite this, the knowledge about the possibility of an interaction is low in Austria. On a population level this knowledge could be taught in schools or via target-group specific information campaigns using picture-based counselling strategies to reach those with the most need. On the health care level, GPs could be an important source of information, at the very least for women taking OCPs. It is hoped that evidence-based guidelines regarding this controversy will become available [34] to support GPs in their counselling as well as patients in their health literacy. For that, GPs should document the use of contraception by their patients; when prescribing antibiotic medications in patients with concomitant OCP use GPs should be aware of this and should inform the patients.
